# The Role of Lipid Metabolism in Dyslipidemias and Atherosclerosis

**DOI:** 10.3390/metabo14110596

**Published:** 2024-11-06

**Authors:** Miodrag Janić, Shizuya Yamashita, Ta-Chen Su, Manfredi Rizzo

**Affiliations:** 1 Department of Endocrinology, Diabetes and Metabolic Diseases, University Medical Centre Ljubljana, 1000 Ljubljana, Slovenia; 2 Faculty of Medicine, University of Ljubljana, 1000 Ljubljana, Slovenia; 3 Department of Internal Medicine and Medical Specialties, School of Medicine, University of Palermo, 90133 Palermo, Italy; manfredi.rizzo@unipa.it; 4 Department of Cardiology, Rinku General Medical Center, Rinku Ourai Kita 2-23, Osaka 598-8577, Japan; s-yamashita@rgmc.izumisano.osaka.jp; 5 Departments of Environmental and Occupational Medicine and Internal Medicine, National Taiwan University College of Medicine, National Taiwan University Hospital, Taipei 10617, Taiwan; tachensu@gmail.com; 6 College of Pharmacy, Ras Al Khaimah Medical and Health Sciences University, Ras Al Khaimah 11172, United Arab Emirates

Atherosclerotic cardiovascular disease is a major burden of morbidity and, despite recent therapeutic advances, is the leading cause of mortality worldwide. It is associated with several risk factors, among them dyslipidemia, arterial hypertension, diabetes, excessive consumption of sugar-sweetened beverages and alcohol, smoking, obesity, and a sedentary lifestyle [1]. The reduction in low-density lipoprotein (LDL) cholesterol, a causal factor for atherosclerosis, by 1 mmol/L results in a 23% reduction in major adverse cardiovascular events (MACE), regardless of how this is achieved [2]. Therefore, dyslipidemia remains a risk factor, where the evident effect of its modulation continues to be demonstrated in different clinical trials. Dyslipidemia is also influenced by other nontraditional risk factors, among which air pollution is probably the one that is now gaining a lot of attention. Other conditions that merit research on their influence on dyslipidemia are also low-grade chronic inflammation, sleep disorders, stress, and psychological factors. These can be only modulators, pathogenetic factors, or therapeutic targets.

Despite the successful treatment strategies available, less than half of our patients are reaching treatment goals. The future of the use of novel and advanced lipid-lowering therapies has already emerged. But one of the biggest challenges in the management of dyslipidemia remains adherence to lifestyle modifications, such as difficulty changing habits and lack of motivation, as well as, particularly, adherence to therapy, as it can have side effects and treatment regimens can be complex [3]. Furthermore, nutraceuticals are sometimes more adhered to than established drugs. In this Special Issue, Luca et al. review lipid-lowering strategies in patients with post-acute coronary syndrome, describing available treatments, evidence on the reduction in cardiovascular risk in this setting, as well as real-world data, guideline recommendations, and discrepancies between the two in achieving guideline-recommended goals. They emphasize the need for novel strategies to quickly and efficiently implement the available lipid-lowering arsenal, as it is already available but is significantly underused in the secondary prevention setting.

As a plethora of data continues to be gathered, even in those who reach target LDL cholesterol levels, residual risk remains, as even these patients still experience cardiovascular events. Yang et al. focused on the assessment of residual cardiovascular risk through lipid abnormalities. They evaluated population concentrations and the distribution of remnant cholesterol in patients hospitalized for acute coronary syndrome, as well as determining the number of patients within the target LDL cholesterol of less than 1.4 mmol/L and non-high-density lipoproteins (HDL) cholesterol of less than 2.6 mmol/L. They found that among patients hospitalized for acute coronary syndrome, 10.9% had still elevated remnant cholesterol levels, defined as ≥1.0 mmol/L, even though they had LDL cholesterol and non-HDL cholesterol within the targets. This finding indicated residual cardiovascular risk, as remnant cholesterol is known to be associated with an increased risk of cardiovascular events. However, challenges remain even in the nonsymptomatic spectrum of patients, calling for effective identification of subclinical dyslipidemia as well as better risk stratification based on genetic predisposition, lifestyle, and other medical conditions.

Additional lipid parameters, in addition to LDL cholesterol, continue to gain popularity, as they may have better specificity and precision in predicting future coronary events. One such parameter is the total-to-HDL cholesterol ratio (TC/HDL) that can reclassify atheroma progression and MACE rates when discordant with LDL cholesterol, non-HDL cholesterol, and apolipoprotein B within patients. In addition, remnant cholesterol is gaining more attention. Lipoprotein subfractions, particularly LDL subfractions, also seem to change due to different therapies, but their determination is still not routine [4]. Furthermore, different lipoprotein subfractions could also predict cardiovascular events, and in this Special Issue, Ye et al. reported the association between coronary artery lesions and a change in plasma lipoprotein subfraction concentrations in patients with unstable angina. They showed that by discriminating lipoprotein subfractions using nuclear magnetic resonance, the plasma lipoprotein profile could be used to distinguish unstable angina patients with a high Gensini score from those with normal coronary arteries. They found that a lipoprotein profile consisting of an increased Apo-B/Apo-A1 ratio and VLDL, as well as a decrease in Apo-A1, LDL-2, LDL-3, HDL, HDL-1, HDL-2, and Apo-A1 was present in patients with unstable angina and a high Gensini score, distinguishing them from people without coronary lesions, while a profile of increased VLDL-4 and decreased LDL-1, HDL, HDL-2, and HDL-3 Apo-A1 and LDL-2 was able to distinguish unstable angina patients from people with normal coronary arteries. The lipoprotein profiles in patients with a low Gensini score and people without coronary artery lesions were similar. They concluded that these lipoprotein subfraction characterizations could be used as biomarkers, add to discrimination of patients, and reclassify them according to different coronary lesions, regardless of four risk factors, i.e., age, sex, body mass index, and use of lipid-lowering drugs.

Genetic testing is gaining increasing recognition in biological pathways that modulate blood lipid levels. But a gap remains between theoretical knowledge and its applicability in clinical practice. There is no defined clinical recommendation for whom genetic testing is indicated. Possible benefits might be in disease confirmation and assessing susceptibility and resistance to treatment. On the other hand, the high cost may be a drawback that makes clinicians reluctant to initiate it. Consequently, genetic testing is probably currently underutilized in clinical practice. However, it should be used selectively, depending on the type of dyslipidemia and when the benefits outweigh the costs [5].

Familial hypercholesterolemia also remains an uncharted territory, as not all people with this state experience MACE in the same share. In this Special Issue, Ganajli et al. evaluated whether the capacity of HDL to efflux cellular cholesterol from lipid-laden macrophages could be a reliable and low-cost biomarker for better risk assessment of premature cardiovascular events in patients with familial hypercholesterolemia, as genetic testing is rarely used due to its high cost. In this study, they report that in patients with homozygous familial hypercholesterolemia, the ability of measured HDL subfractions, HDL-2 and HDL-3, to release cholesterol from lipid-laden macrophages was significantly reduced compared to patients with heterozygous familial hypercholesterolemia and healthy subjects. Their efflux capacity was also inversely associated with homozygous familial hypercholesterolemia. The authors conclude that these findings could identify patients with homozygous familial hypercholesterolemia at increased risk of premature cardiovascular disease.

Furthermore, Koutsogianni et al. focus on familial hypercholesterolemia and its possible association with increased levels of lipoprotein (Lp) (a), both genetically determined entities but not genetically associated. They advocate that Lp (a), particularly among patients with familial hypercholesterolemia, multiplies cardiovascular risk, calling for more population-based studies focusing on the association between the two as well as action for their early detection, as effective therapeutic strategies are available and emerging that could successfully at least in part alleviate this risk.

Although, from the point of view of clinicians, this research provides unprecedented value, a complete understanding of the mechanisms behind blood lipid abnormalities remains unreachable. Investigating the epigenetic mechanisms that contribute to dyslipidemia and exploring potential therapeutic interventions, as well as, for example, determining the role of the gut microbiome in the development of dyslipidemia and its potential as a therapeutic target, are just two of the unanswered questions. Therefore, as reported in this Special Issue, Li et al. propose a new animal model based on polygenic interaction of male B6-Chr1BLD mice for accurate clinical diagnosis and precise medicine of hyperlipidemia. They found that male B6-Chr1BLD mice fed a high-fat diet showed more severe phenotypes related to hyperlipidemia. Several differentially expressed genes enriched for single-nucleotide polymorphisms were involved in lipid metabolism pathways, including *Aida*, *Soat1*, *Scly*, and *Ildr2*. These genes might play an initial and upstream role in the abnormal metabolic phenotype of male B6-Chr1BLD mice. On the other hand, in the setting of insulin resistance and the environment of the so-called atherogenic dyslipidemia, Vekić et al. discuss the effect of advanced glycation end products (AGE) on small, dense LDL cholesterol. AGEs can qualitatively modify small, dense LDL cholesterol, forming the glycated LDL, which is more atherogenic than small, dense LDL per se, thus conferring even more increased cardiovascular risk. The authors discuss the role of glycated LDL and inflammation, particularly in the setting of diabetes mellitus, describing the methods of their determination and the possibilities of their use as guides for therapy selection and risk prediction.

Despite significant advances in research and management of dyslipidemia, as well as some pebbles in the mosaic added with this Special Issue, gaps remain for further investigation. We are in the era of personalized medicine, and therefore the identification of specific genetic variants that influence susceptibility to dyslipidemia and response to treatment remains a challenge. But this will not come at no cost and therefore will not be accessible to all patients. We will need to strive to find suitable pathways to allow more and more patients to benefit from these new possibilities. In addition, finding a suitable drug combination for each patient is also an ongoing area of research. And moreover, the development of novel drug therapies or even vaccines are well underway, and this will in the future require rigorous evaluation in clinical trials. And finally, continuing to identify new lipid abnormalities that contribute to cardiovascular risk will remain a challenge, as will its addition to new and more accurate risk prediction models. Early detection will allow for early and proper intervention with the cumulative goal of reducing cardiovascular risk and ultimately improving quality of life. By addressing these research gaps, we will be able to improve our understanding of dyslipidemia, develop more effective prevention and treatment strategies, and ultimately reduce the burden of cardiovascular disease associated with this condition. But this will require a multifaceted approach that involves healthcare providers, patients, researchers, and policymakers.
